# Biceps Tendon Rupture: Contemporary Evidence, Evolving Techniques, and a Mechanics-to-Management Clinical Framework

**DOI:** 10.7759/cureus.97718

**Published:** 2025-11-24

**Authors:** Mohamed Elgendy, Mahmoud Mersal, Osama Embaby, Sara Elsaidy, Mohamed Alsonbaty

**Affiliations:** 1 Trauma and Orthopaedics, University Hospitals Birmingham (UHB) NHS Foundation Trust, Birmingham, GBR; 2 Orthopaedics, Sandwell and West Birmingham Hospitals NHS Trust, Birmingham, GBR

**Keywords:** biceps tendon rupture, distal biceps repair, fixation techniques, functional outcomes, long head of the biceps, rehabilitation, surgical management, tenodesis, tenotomy

## Abstract

Biceps tendon rupture represents a spectrum of injuries ranging from the degenerative attrition of the long head proximally to acute mechanical failure of the distal insertion. This review synthesises contemporary evidence on anatomy, biomechanics, diagnostic strategies, operative and non-operative management, and long-term outcomes. Proximal ruptures typically occur in hypovascular, degenerative tissue and often coexist with rotator-cuff pathology, whereas distal ruptures result from sudden eccentric overload and produce significant supination and flexion deficits. Diagnosis relies on clinical examination supported by ultrasound or magnetic resonance imaging (MRI) when chronicity or partial tearing is suspected. Management strategies vary by location: proximal ruptures often respond well to tenotomy or tenodesis depending on patient preference, while distal ruptures generally require anatomic repair or graft reconstruction when delayed. Surgical outcomes are excellent when anatomic footprint restoration and proper tensioning are achieved; complications are uncommon and usually transient. Future directions include biologically active fixation, improved imaging biomarkers, and value-based surgical decision-making. Understanding the mechanical and biological differences between proximal and distal biceps pathology remains central to guiding personalised treatment.

## Introduction and background

**Figure 1:**
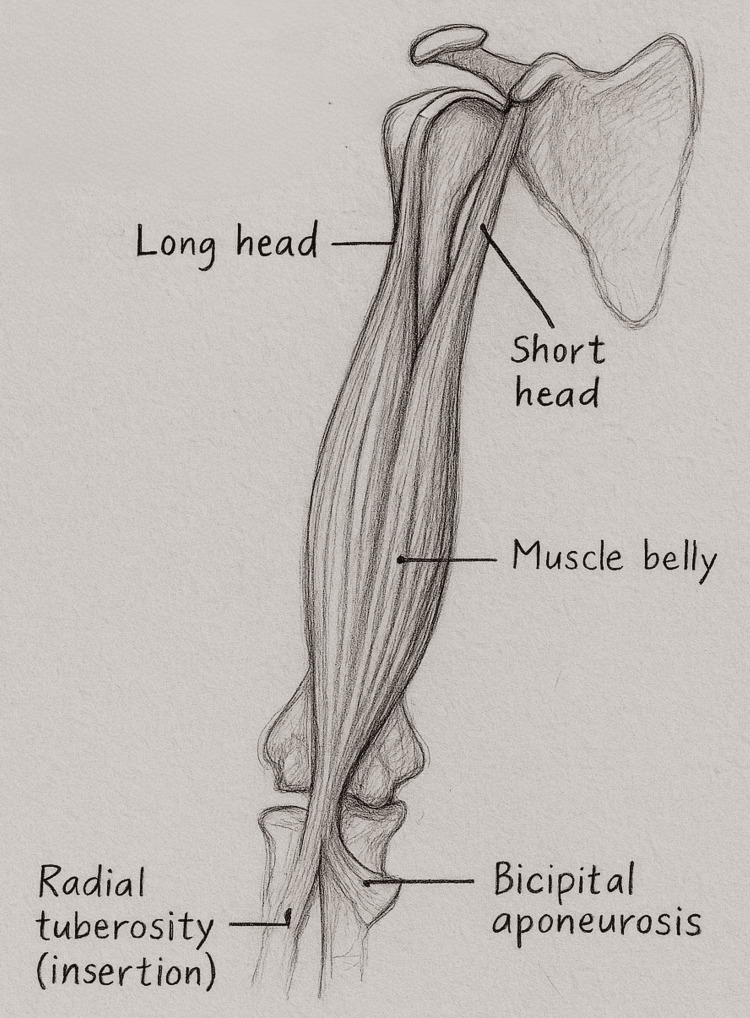
Normal anatomy of the biceps brachii muscle Pencil illustration, originally hand-drawn and created by the authors, showing the normal anatomy of the biceps brachii. The long head originates from the supraglenoid tubercle and the short head from the coracoid process, merging into a single muscle belly that inserts on the radial tuberosity. The bicipital aponeurosis extends medially into the forearm fascia.

The biceps brachii (Figure 1) remains one of the most intriguing muscles in upper limb anatomy, deceptively simple in appearance but remarkably complex in function. It consists of two proximal origins and a single distal insertion, each serving distinct mechanical purposes and exhibiting unique patterns of failure. The long head of the biceps tendon (LHBT) originates from the supraglenoid tubercle and the superior labrum before traversing the rotator-cuff interval and descending through the bicipital groove, while the short head arises from the coracoid process, merging with the muscle belly and contributing to anterior shoulder stability and flexion power [1,2]. Importantly, the LHBT acts as a crucial dynamic stabiliser of the glenohumeral joint against torsional forces, especially when the rotator cuff (supraspinatus and infraspinatus) is compromised, often leading to secondary biceps pathology [3,4]. Distally, both heads unite into a flattened tendon inserting on the posterior aspect of the radial tuberosity, reinforced by the bicipital aponeurosis, which anchors into the forearm fascia [3,4].

Beyond its role in flexion and supination, the LHBT contributes meaningfully to anterior glenohumeral stability. Rodosky et al. demonstrated that tension within the LHBT and superior labral complex restrains anterior humeral head translation during the late cocking phase of overhead motion and acts as an important stabiliser under torsional load [5]. Katthagen et al. further showed that the LHBT resists external rotation torque and anterior shear forces, a stabilising effect that becomes evident particularly when the infraspinatus or subscapularis is compromised, not in isolated supraspinatus deficiency [6]. These findings explain why LHBT degeneration frequently accompanies rotator-cuff insufficiency, especially when posterior or anterior cuff stabilisers fail.

Anatomical and biomechanical research has clarified that the distal insertion is not purely anterior but slightly posterior and ulnar, an orientation that explains the tendon's unique mechanical vulnerability during resisted extension and supination [2]. Hutchinson et al. demonstrated how this oblique footprint predisposes the tendon to eccentric overload [2], while Jarrett and colleagues identified fascicular differences between the two heads: the long head contributes more to supination and the short head more to flexion [3]. Bhatia et al. further showed how subtle forearm rotation can alter the insertional footprint, a nuance critical to anatomic reattachment during surgery [4]. Collectively, these observations highlight why even small deviations in repair tension or orientation can have measurable consequences for strength and motion.

Physiologically, the biceps functions as both a primary supinator and a secondary flexor, working in tandem with the brachialis and brachioradialis. Schmidt et al. quantified how minor variations in reattachment, just a few millimeters, can reduce supination torque substantially [7]. The radial tuberosity itself influences load distribution: Hilgersom and colleagues correlated a larger tuberosity with higher rupture risk, suggesting that increased moment arms create greater shear stress at the tendon-bone interface [8]. At the shoulder, van Deurzen et al. described how a narrow or constricted bicipital groove predisposes the LHBT to attritional wear and tenosynovitis, especially in overhead athletes [9]. Even anatomical variants such as accessory biceps heads, seen in up to 10% of individuals, can modify local biomechanics and alter vector forces across the groove [10].

Vascular anatomy provides another key piece of the puzzle. The intra-articular portion of the LHBT is relatively hypovascular, a "watershed zone" identified by Hufeland et al. and confirmed histologically in multiple mapping studies [11]. Simon et al. found that excised LHBT specimens commonly show myxoid degeneration, fibroblast proliferation, and collagen disorganization, findings consistent with chronic degenerative change rather than inflammation [12]. Simply put, the tendon does not fail because it is inflamed; it fails because it is starved of blood.

This progressive attrition defines the natural course of proximal biceps pathology. Repetitive overhead activity, friction within the groove, and shear forces from rotator-cuff imbalance gradually lead to fraying, tearing, and eventual rupture [13]. The link between rotator-cuff disease and LHBT degeneration is well established. Walch et al. and others demonstrated that up to 70% of massive cuff tears involve concomitant biceps pathology [14,15]. In such cases, the long head is not the initiator but the victim, an overworked stabiliser in a failing shoulder complex.

Distal biceps rupture, on the other hand, represents a completely different mechanism. It typically affects middle-aged men during a sudden eccentric contraction, most often while lifting or catching a falling object [16,17]. The incidence is estimated at two to five cases per 100,000 person-years, overwhelmingly involving the dominant arm [18,19]. Risk factors such as smoking, steroid use, and obesity significantly weaken tendon collagen, increasing susceptibility to rupture [16]. Lappen et al. used high-speed video analysis to show that rupture commonly occurs when the elbow is nearly extended and the forearm supinated, precisely the position that maximises tensile load at the radial tuberosity [17].

Functionally, the consequences are immediate. Studies have demonstrated a 40% loss of supination and 30% loss of flexion strength following untreated rupture, underscoring why active individuals rarely tolerate conservative management [20]. Fortunately, modern anatomic repair techniques restore near-normal power when the native footprint and tendon orientation are respected [21].

Thus, the biceps tendon embodies two distinct failure models within one muscle: degenerative attrition proximally and acute mechanical failure distally. Both arise from the same finely tuned anatomy, a structure designed for efficiency but unforgiving of abuse. Understanding its form and physiology is therefore the foundation for rational treatment; every surgical decision, from tenodesis site to fixation angle, is ultimately a conversation with the tendon's own design.

## Review

Pathophysiology and mechanisms

The biceps tendon's twin-headed design produces two entirely different modes of failure. Proximally, the long head degenerates gradually under repetitive attrition and poor vascularity; distally, the tendon fails abruptly under sudden eccentric overload. However, both share the same biological prelude, microscopic collagen degeneration that silently weakens the structure until a final catastrophic event occurs.

Proximal long-head degeneration

Histological and vascular studies have shown that the LHBT undergoes chronic ischemic degeneration rather than acute inflammation. Hufeland et al. mapped a distinct hypovascular zone within the intra-articular segment, precisely at the common rupture site [11]. Simon et al. later confirmed these findings, demonstrating myxoid degeneration, fibroblast proliferation, and collagen disorganisation in excised LHBT specimens, features typical of chronic tendinopathy rather than tendonitis [12]. These observations shifted our understanding of LHBT disease from an inflammatory to a degenerative process.

**Figure 2:**
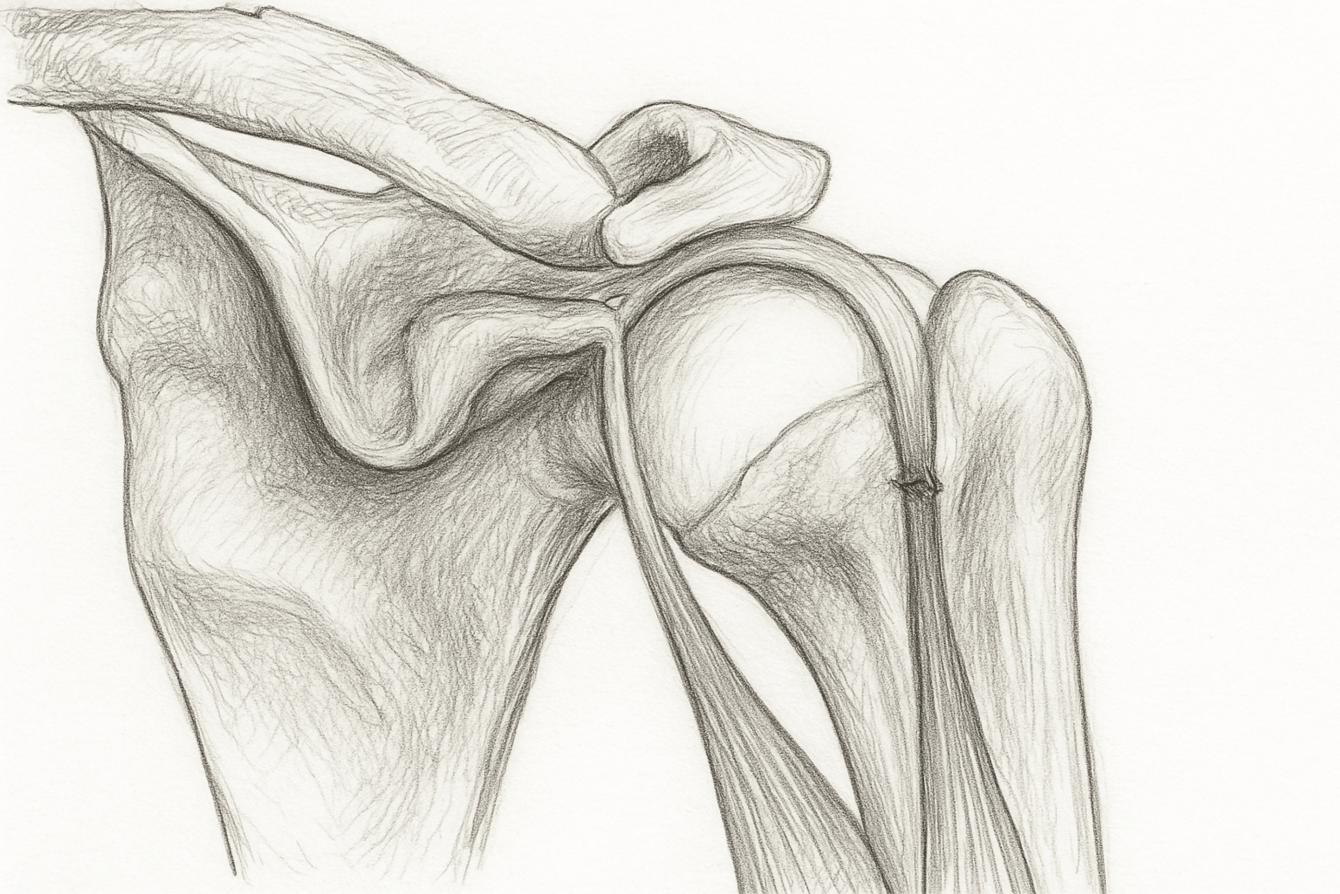
Proximal long head biceps tendon rupture Pencil illustration, originally hand-drawn and created by the authors, showing a proximal long head biceps tendon rupture. The drawing depicts the detachment of the long head from the supraglenoid tubercle and the retraction of the muscle belly, while the short head remains intact at the coracoid process.

Mechanical stress further accelerates this attrition. As the tendon glides through the bicipital groove (Figure 2), it endures constant torsion and compression. van Deurzen et al. showed that a narrowed or irregular groove heightens frictional wear [9], while Gheno et al. described accessory biceps heads that alter the line of pull and local tension [10]. Repetitive shear promotes the delamination of collagen fibers and gradual elongation. Once the transverse humeral and superior glenohumeral ligaments fail, the tendon subluxates medially, worsening degeneration.

However, not all studies support groove morphology as a primary driver of LHBT pathology. Cardoso et al. found no statistically significant association between bicipital-groove cross-sectional measurements and intra-articular LHBT lesions, suggesting that groove anatomy alone does not predict disease [22]. Beall et al. reported that LHBT pathology shows a stronger correlation with anterosuperior rotator-cuff tears, particularly supraspinatus and subscapularis involvement, than with isolated groove features, reinforcing the concept that LHBT injury often reflects a broader failing shoulder complex rather than isolated mechanical impingement [23].

This pathology rarely exists in isolation. Walch et al. and Chen et al. demonstrated that up to 70% of massive rotator-cuff tears are accompanied by LHBT disease [14,15]. As the cuff weakens, the biceps assumes a compensatory stabilising role, further overloading the tendon. Consequently, proximal rupture often represents the final stage of a failing shoulder complex rather than a solitary injury. In many cases, patients experience pain relief after tenotomy or tenodesis because the tendon's removal eliminates a secondary pain generator rather than sacrificing a vital structure [12,13].

Distal tendon failure

The distal tendon fails in a completely different environment, one of acute mechanical overload. Peak supination torque occurs around 90° of elbow flexion, but when the elbow suddenly extends against resistance, stress at the radial tuberosity footprint surges beyond the tendon's tolerance [3,4,8]. Lappen et al. used motion analysis to confirm that rupture typically occurs with the elbow nearly extended and the forearm supinated [17]. The tendon's posterior-ulnar insertion, first detailed by Hutchinson et al., explains why this motion produces a tangential shear vector that lifts rather than tears the tendon cleanly [2].

Even so-called "acute" ruptures rarely arise in perfectly healthy tissue. Safran and Graham identified smoking, steroid use, and metabolic disease as major risk factors, each of which weakens collagen integrity [16]. Hilgersom et al. showed that individuals with a larger radial tuberosity paradoxically face higher rupture risk, likely because the increased moment arm magnifies torque at the insertion [8]. The distal tendon's fascicular junction between long- and short-head fibers may represent an additional intrinsic weak point [3].

The transition from micro- to macro-failure

Regardless of location, rupture represents the endpoint of a single biological continuum: collagen disorganisation → mucoid degeneration → fascicular delamination → complete discontinuity. Recent imaging advances support this sequence. Loegering et al. demonstrated with ultrashort-echo-time magnetic resonance imaging (MRI) that microstructural signal alterations precede visible tearing, suggesting the potential for early detection [24]. Though not yet clinically routine, such findings could pave the way for pre-emptive interventions in high-risk patients.

Functionally, distal rupture leads to an immediate and measurable deficit, roughly 40% loss of supination and 30% loss of flexion torque if left unrepaired [20]. Proximal rupture, by contrast, alters cosmetic contour with minimal strength loss [13]. This physiological distinction underpins the contrasting management philosophies: distal ruptures warrant anatomical repair, and proximal lesions can often be treated conservatively or with simple tenodesis.

Thus, the biceps tendon's very sophistication is its weakness, a long, narrow, and hypovascular cable engineered for precision rather than abuse. Proximal failure is the result of slow ischemic wear, but distal failure is an instant mechanical overload. Any modern management strategy must begin with that fundamental understanding.

Diagnosis and clinical evaluation

Diagnosis of biceps tendon pathology requires integrating anatomical insight with clinical pattern recognition. Although the physical signs are often obvious, subtle differences between proximal and distal injuries, and between acute and chronic states, demand a systematic, experienced approach.

Clinical Presentation: Proximal vs Distal

**Figure 3:**
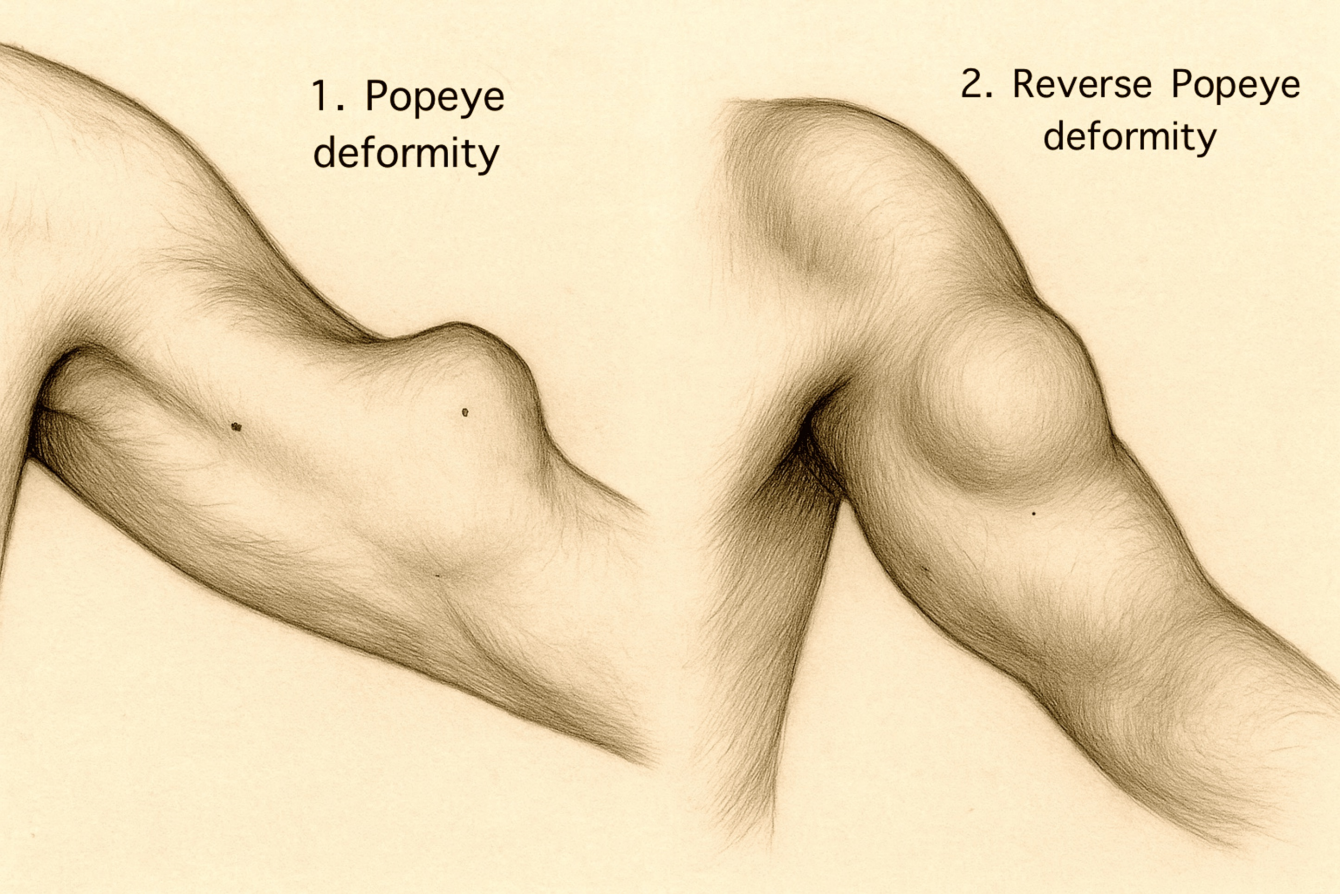
Popeye and reverse Popeye deformities Pencil illustration, originally hand-drawn and created by the authors, demonstrating the classic and reverse forms of biceps deformity. The image on the left shows the characteristic distal bulge of the muscle belly following the long proximal biceps head rupture ("Popeye deformity"), while the image on the right depicts the proximal retraction of the biceps muscle following the rupture of the distal biceps tendon ("reverse Popeye deformity").

Proximal ruptures usually present with the classic "Popeye" deformity (Figure 3), a mid-arm bulge following an audible snap or tearing sensation [13]. Pain often subsides quickly, leaving a cosmetic defect and mild endurance weakness. Because the short head remains intact, most patients maintain near-normal elbow strength [1,3]. In many cases, the rupture brings relief rather than disability, particularly in patients with pre-existing cuff pathology [12].

Distal ruptures, conversely, produce a "reverse Popeye" deformity with flattening of the antecubital fossa (Figure 3), ecchymosis extending distally, and notable weakness in supination and flexion [13,16,25]. Morrey et al. and Carrazana-Suarez et al. quantified these deficits at roughly 40% and 30%, respectively [20,26]. Thus, while proximal rupture primarily affects form, distal rupture compromises function.

Physical Examination

For distal injuries, O'Driscoll's Hook Test remains the most reliable maneuver; the inability to hook the tendon with the index finger while the elbow is flexed and supinated indicates complete avulsion [27]. Ruland et al. later described the Biceps Squeeze Test, where compression of the biceps belly fails to elicit forearm supination in complete tears [28]. Luokkala et al. showed that both tests retain high sensitivity in acute cases but decline in accuracy after three weeks [29].

Partial tears are more elusive. Williams et al. found that tenderness over the radial tuberosity with preserved but painful supination should prompt MRI evaluation [30]. Devereaux and ElMaraghy demonstrated that combining the Hook and Squeeze tests raises sensitivity to nearly 98% [31].

For proximal lesions, the Speed and Yergason tests have limited specificity [32]. Pain over the groove during resisted flexion or supination suggests LHBT involvement but often coexists with rotator-cuff disease. Palpation of the bicipital groove and observation of medial subluxation during rotation remain the most revealing clinical clues [9,13] (Table 1).

**Table 1:** Clinical evaluation of biceps pathology The table summarises the key physical examination maneuvers used to differentiate proximal LHBT pathology from complete distal biceps tendon rupture, including their target pathology and reported approximate diagnostic performance (sensitivity and specificity). LHBT: long head of the biceps tendon

Test	Target pathology	Positive finding	Sensitivity (%)	Specificity (%)	Key reference(s)
Speed's Test	Proximal (LHBT) tendinopathy	Pain in bicipital groove during resisted forward flexion of the shoulder	≈32-63%	≈58-75%	Krupp et al. [32]
Yergason's Test	Proximal (LHBT) pathology (subluxation, tendinopathy)	Pain or click in bicipital groove during resisted forearm supination/external rotation	≈32-43%	≈78-79%	Krupp et al. [32]
Hook Test	Distal (complete rupture)	Inability to "hook" the distal tendon with the index finger	≈80-100% (acute complete tears)	≈96-100% (acute)	O'Driscoll et al. [27], Luokkala et al. [29]
Biceps Squeeze Test	Distal (complete rupture)	Lack of forearm supination upon compression of the biceps muscle belly	≈96% alone, ≈98% combined with Hook Test	High (limited data; few false positives reported)	Ruland et al. [28], Devereaux and ElMaraghy [31]

Imaging

Imaging refines diagnosis, clarifies chronicity, and aids surgical planning. Ultrasound is the frontline tool. Dynamic scanning can identify subluxation, tenosynovitis, or focal disruption. Lobo et al. reported over 90% sensitivity for proximal and 95% for distal complete ruptures when performed by experienced sonographers [33].

MRI remains the reference standard. In proximal disease, MRI delineates groove morphology, labral attachment, and associated cuff pathology [9,13]. In distal tears, T2-weighted and proton-density sequences reveal tendon retraction and the "empty tuberosity" sign. Luokkala et al. confirmed its reliability when physical findings were equivocal [29]. Loegering et al. further showed that ultrashort-echo-time MRI detects micro-degenerative changes before rupture [24].

Computed tomography (CT) or MR arthrography may assist in revision or postoperative settings, while dynamic ultrasound uniquely visualises an unstable LHBT subluxating within the groove [33,34].

Chronic and Atypical Presentations

Chronic distal ruptures may present subtly once bruising resolves. The Hook Test may be falsely negative, so MRI becomes essential to evaluate retraction and tissue viability [25,29,33]. These patients often complain of endurance fatigue rather than acute pain. Early recognition determines whether direct repair remains possible or if graft reconstruction is required [35,36].

Chronic proximal pathology often mimics impingement or cuff disease for months before rupture. High-resolution MRI helps distinguish partial attrition from complete tearing, imaging findings that mirror the histologic degeneration described by Simon et al. [12].

Integrating Clinical and Imaging Findings

The art of diagnosis lies in synthesis. Physical examination offers functional insight, and imaging confirms anatomy and chronicity. In distal rupture, a positive Hook Test coupled with supination weakness and MRI confirmation provides near-absolute diagnostic certainty [27,29,31].

In proximal pathology, a Popeye deformity with groove tenderness and cuff-related pain is almost pathognomonic. Imaging defines the extent, not the existence, of the lesion. Ultimately, experienced clinicians diagnose biceps tendon rupture with their hands and eyes before the scanner ever starts. Technology informs judgment; it does not replace it.

Management strategies

The management of biceps tendon pathology hinges as much on purpose as on anatomy. What matters is not only what has torn but who it has torn in. A spontaneous rupture that frees an elderly patient from chronic shoulder pain may cripple a young athlete who lives by his grip. Treatment, therefore, begins with context, not the scalpel.

Non-operative Management

Proximal long-head rupture is usually forgiving. In most older or low-demand patients, comfort and function return with simple physiotherapy. Simon et al. showed that pain reduction after spontaneous LHBT rupture mirrors that seen after surgical tenotomy [12]. The only trade-offs are the cosmetic Popeye bulge and occasional mild cramping. Rehabilitation should target shoulder motion, rotator-cuff strength, and scapular control rather than the biceps itself.

Distal rupture, in contrast, is a different story. Morrey et al. reported that conservative management results in roughly 30-40% loss of supination and 30% loss of flexion strength [20]. While sedentary or medically frail patients may adapt, anyone relying on upper limb power for work or sport rarely tolerates this deficit. Physiotherapy maintains motion but cannot restore torque, and patients should understand this distinction clearly before declining surgery.

Operative Management of Distal Ruptures

Timing of repair:** **Early repair, ideally within the first three weeks, remains the technical sweet spot, allowing for direct anatomic reattachment before scarring and retraction complicate mobilization. The tendon is pliable, the musculotendinous junction is mobile, and fixation can be achieved with minimal tension.

However, delay is not a contraindication. Modern fixation techniques have made it possible to achieve strong outcomes even beyond the acute period. Savin et al. demonstrated that delayed repairs can yield comparable strength and function to early surgery, though with a slightly higher risk of transient lateral antebrachial cutaneous-nerve neuropraxia [37]. The rule is simple: repair early if you can, but repair well whenever you do.

When the tendon cannot be advanced to the radial tuberosity despite mobilization, graft reconstruction becomes essential. Hendy et al. reported excellent outcomes using semitendinosus and Achilles allografts, achieving near-normal supination strength with minimal rerupture risk [35]. Frank et al. confirmed these findings, showing that graft reconstruction produces functional results equivalent to acute repair when tension is properly restored [36]. The decisive factor is not time, but tension; restoring the physiologic arc of force is what determines success.

Surgical approaches:** **The century-old argument between single-incision and double-incision exposures has mellowed into consensus: both work well when done properly. Amin et al. reviewed over a thousand distal biceps repairs and found no significant difference in final strength or patient-reported outcomes between the two approaches [38]. Castioni et al. echoed these results, noting that the single-incision technique carries a slightly higher incidence of transient neurapraxia, while the double-incision approach carries a small risk of heterotopic ossification or radioulnar synostosis [39].

In practice, the incision matters far less than the precision. The key is to maintain gentle subperiosteal dissection, pronate the forearm to protect the posterior interosseous nerve, and secure the tendon in its anatomic footprint. The approach should match the surgeon's skillset, not ideology.

Fixation techniques:** **Biomechanical studies consistently place cortical-button fixation at the top for pull-out strength and cyclic stability. Forlenza et al. demonstrated that intramedullary cortical-button constructs provide superior mechanical properties compared with suture anchors or interference screws, though this superiority seldom translates into clinically measurable differences [40]. Wörner et al. later found that partial tears repaired with anchor-only fixation showed higher failure rates than those repaired with Endobutton constructs [41].

The message from these studies is straightforward: anatomic footprint restoration, not implant branding, dictates recovery. Provided the tendon is reattached at its correct orientation and tension, any contemporary fixation can succeed [37].

**Figure 4:**
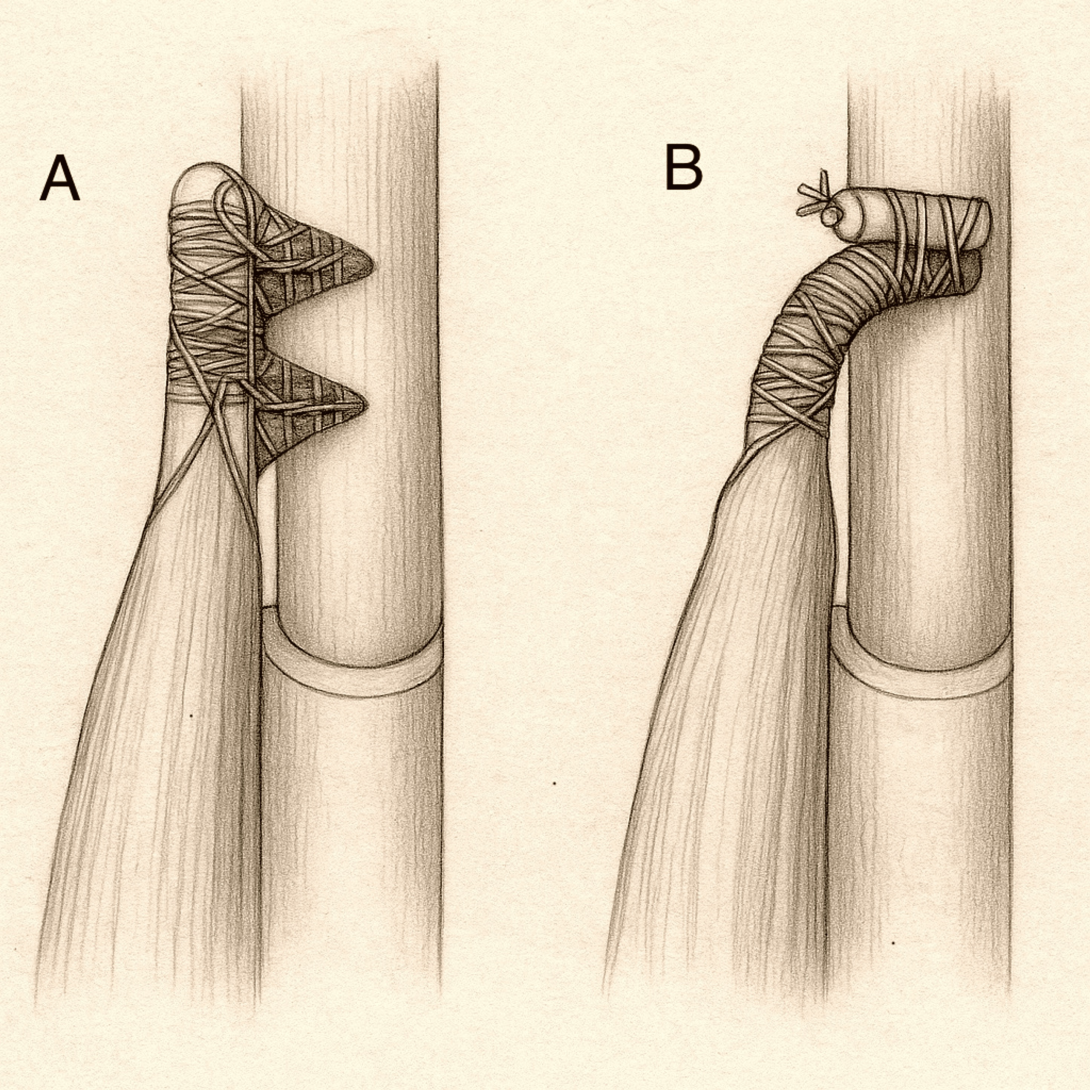
Inlay and onlay techniques for distal biceps tendon repair Pencil illustration, originally hand-drawn and created by the authors, comparing the two primary methods of distal biceps tendon reattachment. Panel (a) demonstrates the inlay technique, where the distal biceps tendon is docked into a bone trough within the radial tuberosity and fixed using sutures or cortical buttons. Panel (b) shows the onlay technique, where the tendon is laid over the cortical surface of the tuberosity and fixed externally.

Inlay vs onlay fixation: A growing point of interest is the distinction between inlay and onlay reattachment. In inlay fixation (Figure 4), the tendon is seated into a prepared bone trough or socket within the radial tuberosity, providing broad contact and more anatomic orientation. This approach better replicates the native footprint and optimises tendon-to-bone healing.

Conversely, onlay fixation (Figure 4) secures the tendon on the cortical surface with suture anchors, a technically simpler method that avoids deep drilling but positions the tendon slightly anterior to its natural insertion. This may modestly alter the supination moment arm, although its clinical impact remains minimal.

Savin et al. confirmed that restoring the tendon's native footprint, whether via inlay or onlay, is more important than the choice of implant [37]. Biomechanical studies such as those by Ernstbrunner et al. found no significant difference in postoperative torque or function between the two methods when tension was appropriate [42].

Thus, the practical philosophy is clear: use inlay fixation when exposure and bone quality allow, and use onlay when simplicity or anatomy dictates. In both, precision of placement trumps device preference.

Postoperative rehabilitation: Rehabilitation philosophy has shifted dramatically over the past two decades, from rigid immobilization toward early, guided motion. The modern principle is simple: stable fixation allows safe movement, and movement fosters recovery.

Early studies that advocated six weeks of immobilization were born of weaker fixation constructs. With the advent of cortical buttons, hybrid anchors, and stronger sutures, immediate controlled motion is now both possible and safe. Huynh et al. showed that compliance with rehabilitation, not the length of immobilization, is the strongest predictor of functional outcome [43].

The pragmatic sequence followed in most contemporary centers is as follows: weeks 0-1 (elbow protected in 90° flexion using a sling or brace; active shoulder, wrist, and hand motion encouraged), weeks 2-6 (begin active-assisted and gravity-eliminated elbow motion; avoid resisted supination), and weeks 6-12 (progressive resistance and strengthening, focusing on endurance and proprioception). After 12 weeks, patients can gradually return to full activity or sport once a pain-free full range of motion and near-symmetrical strength compared with the contralateral side have been achieved.

Biology, not the calendar, sets the pace. A patient with secure fixation but poor tissue quality still heals slower than a young athlete. Protocols must be individualised rather than templated. Over-zealous motion leads to laxity; over-caution leads to stiffness. The art lies in balance and in consistent patient education.

Operative Management of Proximal (Long-Head) Ruptures

Tenotomy vs tenodesis:** **Both operations relieve pain effectively, but they suit different patients and temperaments. Tenotomy is quick, low-risk, and favored in older or sedentary individuals who are less concerned about contour. Tenodesis, though technically more demanding, offers better cosmesis and less cramping, ideal for younger or active patients.

Multiple level-I trials have proven their equivalence in pain relief and function. Belk et al. [44], MacDonald et al. [45], and Na et al. [46] all found no significant difference in postoperative scores or strength. Kooistra et al. [47] and Anil et al. [48] confirmed this across large meta-analyses, concluding that the only consistent advantage of tenodesis lies in reduced Popeye deformity rates (≈6% vs 20% after tenotomy).

More recently, Tu et al. [49] conducted a prospective randomised controlled trial (RCT) comparing open subpectoral and arthroscopic suprapectoral tenodesis. Both achieved near-identical strength recovery and satisfaction scores at one year. These findings reinforce that pain relief is universal and perfection of contour is optional.

Histologic and imaging studies add nuance: Simon et al. [12] demonstrated that the resected LHBT almost always shows degenerative change, validating tenotomy or tenodesis as true disease-removal procedures rather than cosmetic operations. The choice, therefore, should rest on lifestyle and expectation, not ideology.

Subpectoral vs suprapectoral tenodesis:** **The discussion has shifted from "which is better" to "which suits the case". Subpectoral fixation allows the full removal of degenerated tendon and permits robust interference-screw or cortical-button fixation, but it requires a slightly longer incision and carries a small risk of axillary-nerve traction. However, suprapectoral fixation, performed arthroscopically or mini-open, preserves the proximal sheath and minimises scarring but may leave behind frayed intra-groove fibers, which can perpetuate discomfort.

A 2024 meta-analysis by van Deurzen et al. [50] found no meaningful difference in functional outcomes, reoperation rates, or complication profiles between the two techniques. What mattered most was achieving physiologic tension; overtightening causes anterior shoulder pain, and undertensioning reproduces the Popeye deformity.

Biomechanical evaluation by Millett et al. [51] and Urch et al. [52] further supported that both fixation sites provide similar tendon-to-bone healing, provided secure fixation and gradual loading are respected. Recent data by Hassan et al. [53] added that patients' perceived satisfaction correlates more with preoperative education and expectation management than with surgical technique itself.

In short, the best tenodesis is the one the surgeon performs consistently and well. Both methods work; the difference lies in execution, not incision.

Rehabilitation after proximal procedures:** **Rehabilitation after tenodesis mirrors that of distal repair in principle but with lighter loading. Passive- and active-assisted shoulder motion starts immediately, avoiding resisted elbow flexion and supination for the first four to six weeks. Strengthening follows once pain-free motion returns, and full recovery typically occurs by three months [54].

As with distal injuries, the greatest determinant of success is compliance. Patients who engage with physiotherapy regain full function faster, while those who self-limit remain stiff or weak despite technically perfect surgery.

Table 2 summarises the management strategies for biceps tendon rupture.

**Table 2:** Summary of management strategies for biceps tendon rupture The table provides a high-level comparison of non-operative and operative management strategies, stratified by pathology (proximal vs distal) and patient factors (demand/activity level). LHBT: long head of the biceps tendon

Pathology	Patient profile	Primary non-operative	Primary operative	Key considerations
Proximal (LHBT)	Low-demand, elderly	Physiotherapy, observation	Tenotomy (simple, cosmetic defect)	Pain relief is excellent for both. Tenodesis avoids "Popeye" deformity [44,47]
	High-demand, young, cosmetic concern	N/A	Tenodesis (subpectoral or suprapectoral)	-
Distal (tendon)	Low-demand, frail	Physiotherapy (accepts weakness)	N/A	Non-operative results in significant strength loss (30% flexion, 40% supination) [20]
	High-demand, active	N/A	Surgical repair (button, anchor, etc.)	Early repair is ideal. Graft reconstruction is effective for chronic tears [35,36]

Outcomes, complications, and future directions

Functional Outcomes

Across all forms of biceps tendon injury, outcomes depend far more on indication and surgical precision than on the device or approach. When diagnosis is correct and technique sound, the results are consistently excellent.

In distal repairs, operative treatment restores over 90% of contralateral torque and achieves return-to-work rates above 95% [21]. Even in chronic cases, provided the tendon is reconstructed under proper length-tension balance, functional recovery is near-complete [35,36]. Both Henry et al. [55] and Forlenza et al. [40] documented rerupture rates below 2% and uniformly high satisfaction across fixation types. Vanderlinden et al. [21] further confirmed that Endobutton fixation via a single anterior approach maintains durable strength and low complication rates beyond five years.

In proximal pathology, the story is equally reassuring. Belk et al. [44] and MacDonald et al. [45] confirmed that pain relief and shoulder function scores are equivalent between tenotomy and tenodesis, with the only real difference being cosmetic. Tu et al. [49] and van Deurzen et al. [50] demonstrated that both subpectoral and suprapectoral techniques restore full function in the majority of patients, validating surgeon preference as the decisive factor rather than technique. Long-term series by Kooistra et al. [47] reinforced these findings, noting persistent improvement in strength and comfort even among elderly cohorts.

Complications

Complications are relatively uncommon, but they remain some of the best teachers in orthopaedics. After distal repair, the most frequent issue is the transient irritation of the lateral antebrachial cutaneous nerve, occurring in roughly 9% of single-incision cases [38]. This typically resolves spontaneously within weeks. Posterior interosseous-nerve palsy, the most feared but least common event, remains below 2% with modern pronation-based protection techniques [25].

Pre-existing double-incision approaches, once notorious for heterotopic ossification, now show sharply reduced rates, thanks to meticulous subperiosteal dissection and short-course non-steroidal anti-inflammatory drug (NSAID) prophylaxis [25,39]. Fixation failures are increasingly rare (<1%), but overtensioning or malpositioning of the tendon can produce chronic forearm discomfort or dysesthesia, as highlighted by Castioni et al. [39].

After proximal tenodesis, the leading complaints are mild groove pain or subpectoral tenderness, seen in around 3% of patients, which nearly always resolves with physiotherapy [50]. Tenotomy carries a 15-20% incidence of cosmetic "Popeye" deformity, but this rarely impairs strength or satisfaction [44,47]. In fact, patient-reported outcomes suggest that cosmetic dissatisfaction is often overestimated by surgeons and underweighted by patients [53].

Predictors of Poor Outcome

Even with perfect surgery, biology and behavior can still determine failure. Safran and Graham [16] identified smoking and anabolic-steroid use as independent predictors of rupture and impaired tendon healing. Hilgersom et al. [8] found that individuals with larger radial tuberosity morphology were more prone to distal rupture due to altered mechanical stress. Persistent hypovascularity, as documented by Hufeland et al. [11], likely contributes to incomplete tendon-bone integration after repair.

Perhaps the most underappreciated variable is patient compliance. Huynh et al. [43] demonstrated that adherence to rehabilitation directly predicts functional recovery; a compliant patient with modest fixation often outperforms a noncompliant one with flawless hardware.

Long-Term Outcomes and Quality of Life

The long-term picture is one of durable success. Launonen et al. [19] reported a steady rise in surgical incidence across Scandinavia over two decades without a corresponding increase in complication rates. Vanderlinden et al. [21] and Bhatia et al. [4] confirmed that anatomic reinsertion preserves native forearm mechanics and strength even beyond five years.

In proximal disease, Na et al. [46] and Kooistra et al. [47] observed sustained pain relief and functional improvement, including in elderly and low-demand populations. A recent 2024 follow-up by Tagliero et al. [56] documented that over 90% of patients maintained their postoperative gains at 10 years, underscoring the durability of modern repair techniques.

Current Controversies and Evidence Gaps

Despite the high success rates, several debates continue to simmer quietly in surgical circles. 

Timing of repair: Although early repair simplifies exposure, delayed repair with graft augmentation can achieve equivalent outcomes [35,36].

Fixation superiority: Cortical-button constructs dominate biomechanics, but clinical outcomes equalise once proper tension and rehabilitation are achieved [21,37,40,55].

Tenodesis site: Subpectoral versus suprapectoral fixation remains largely preference-driven, with outcomes governed more by tensioning than by location [49-51].

The frontier now lies in biologic augmentation and imaging innovation. Longo et al. [57] reviewed tendon augmentation grafts demonstrating histologic improvements in tendon-to-bone integration in some experimental and clinical settings, while Urch et al. [52] showed that tendon-to-bone tenodesis produces more favorable healing than tendon-to-tendon repair in a rat model. However, these biologic and technical advances have not yet translated into consistently superior clinical outcomes.

On the diagnostic side, Carrazana-Suarez et al. [26] visualised early intratendinous degeneration on MRI, while Lin et al. [58] leveraged deep learning algorithms to diagnose and classify rotator-cuff tears on shoulder MRI with over 90% accuracy, illustrating how similar techniques could eventually be applied to long-head biceps degeneration, given the strong association between cuff tears and LHBT pathology [23].

Economic considerations are also entering the conversation. Neary et al. [59] showed that, in ankle syndesmotic injuries, suture-button constructs are more expensive than screws but may be more cost-effective when reoperation rates are considered. By analogy, similar cost-utility thinking is likely to shape implant choice in distal biceps repair, where biomechanical gains from more complex constructs must be weighed against higher cost for non-elite patients.

Future Directions

The next evolution of biceps-tendon surgery will not be driven by new hardware, but by precision, personalization, and biologic integration. "Smart fixation", implants that combine mechanical stability with biologically active surfaces, is moving from laboratory to clinic. Govindaraju et al. [60] demonstrated that nanofiber scaffolds can promote tendon-to-bone regeneration, suggesting a future where implants facilitate true enthesis restoration rather than simple reattachment.

Furthermore, the role of biologic augmentation remains controversial. While laboratory studies show promise, recent meta-analyses on the use of platelet-rich plasma (PRP) in both distal biceps repair and proximal tenodesis have failed to demonstrate any significant improvement in clinical outcomes, healing rates, or strength compared to repair alone [61,62].

Experimental and review data suggest that tendon augmentation grafts and optimised tendon-to-bone healing may enhance the biologic interface [52,57]. However, high-quality meta-analyses of PRP augmentation around the shoulder and elbow have not demonstrated consistent improvements in pain, function, or rerupture rates [61,62].

Equally, rehabilitation is becoming digitalised. Wearable sensors and app-based motion tracking are being integrated into recovery protocols, allowing the objective monitoring of progress and compliance.

The research agenda ahead is clear: multicenter randomised trials that integrate functional outcomes, patient satisfaction, and cost-effectiveness, not just torque ratios. Until such data emerge, the guiding virtues remain timeless, anatomy, restraint, and craftsmanship.

## Conclusions

Biceps tendon injuries may seem straightforward, but their management still depends on judgment more than hardware. The anatomy teaches humility; what works for one patient fails for another. The real skill lies in restoring balance, not just reattaching tissue. Whether treated with tenodesis, tenotomy, or anatomic repair, success comes from precision, patience, and respect for biology. In the end, good surgery simply gives the tendon back what time or trauma took away, its function and quiet strength.
